# Regulation of the apoptosis-inducing kinase DRAK2 by cyclooxygenase-2 in colorectal cancer

**DOI:** 10.1038/sj.bjc.6605144

**Published:** 2009-07-28

**Authors:** G A Doherty, S M Byrne, S C Austin, G M Scully, D M Sadlier, T G Neilan, E W Kay, F E Murray, D J Fitzgerald

**Affiliations:** 1 Conway Institute of Biomolecular and Biomedical Research, University College Dublin, Dublin, Ireland; 2 Department of Clinical Pharmacology, Royal College of Surgeons in Ireland, Dublin, Ireland; 3 Department of Gastroenterology, Beaumont Hospital and Royal College of Surgeons in Ireland, Dublin, Ireland; 4 Department of Histopathology, Beaumont Hospital and Royal College of Surgeons in Ireland, Dublin, Ireland

**Keywords:** colorectal cancer, cyclooxygenase-2, prostaglandin, NSAIDs

## Abstract

**Background:**

Cyclooxygenase-2 (COX-2) is over-expressed in colorectal cancer (CRC), rendering tumour cells resistant to apoptosis. Selective COX-2 inhibition is effective in CRC prevention, although having adverse cardiovascular effects, thus focus has shifted to downstream pathways.

**Methods:**

Microarray experiments identified genes regulated by COX-2 in HCA7 CRC cells. *In vitro* and *in vivo* regulation of DRAK2 (DAP kinase-related apoptosis-inducing kinase 2 or STK17*β*, an apoptosis-inducing kinase) by COX-2 was validated by qRT-PCR.

**Results:**

Inhibition of COX-2 induced apoptosis and enhanced DRAK2 expression in HCA7 cells (4.4-fold increase at 4 h by qRT-PCR, *P*=0.001), an effect prevented by co-administration of PGE_2_. DRAK2 levels were suppressed in a panel of human colorectal tumours (*n*=10) compared to normal mucosa, and showed inverse correlation with COX-2 expression (*R*=−0.68, *R*^2^=0.46, *P*=0.03). Administration of the selective COX-2 inhibitor rofecoxib to patients with CRC (*n*=5) induced DRAK2 expression in tumours (2.5-fold increase, *P*=0.01). *In vitro* silencing of DRAK2 by RNAi enhanced CRC cell survival following COX-2 inhibitor treatment.

**Conclusion:**

DRAK2 is a serine–threonine kinase implicated in the regulation of apoptosis and is negatively regulated by COX-2 *in vitro* and *in vivo*, suggesting a novel mechanism for the effect of COX-2 on cancer cell survival.

Colorectal cancer (CRC) remains a leading cause of cancer death, with worldwide one million new cases each year and as many as half a million cancer deaths annually (Boyle and Leon, 2002). Cyclooxygenase-2 (COX-2) expression is increased in the majority of colorectal tumours (Eberhart *et al*, 1994) and this induction is associated with advanced tumour stage and correlates with poor clinical outcomes (Sheehan *et al*, 1999). Non-steroidal anti-inflammatory drugs (NSAIDs), which inhibit COX activity, show anti-neoplastic effects *in vitro* (Richter *et al*, 2001; Sheng *et al*, 1997) and human studies have demonstrated their use to be associated with a reduced incidence of colorectal neoplasia (Baron *et al*, 2003; Sandler *et al*, 2003). Although more recent studies have confirmed the chemopreventive activity of COX-2-selective NSAIDs (Arber *et al*, 2006; Baron *et al*, 2006; Bertagnolli *et al*, 2006), it is also clear that their long-term use is associated with an unacceptable increase in the risk of cardiovascular events (Bertagnolli *et al*, 2006; Bresalier *et al*, 2005).

The anti-neoplastic properties of these agents result from the inhibition of prostaglandin generation, particularly that of prostaglandin E_2_ (PGE_2_), the most abundant *in vivo* product of COX-2 activity in CRC cells (Pugh and Thomas, 1994; Rigas *et al*, 1993). Although it appears that PGE_2_ modulates various processes that are fundamental to tumour cell survival, such as altered proliferation and susceptibility to apoptosis (Sheng *et al*, 1997, 1998, 2001; Tang *et al*, 2002), the precise molecular mechanisms remain unclear. A strong rationale exists therefore to generate a more complete understanding of the downstream targets of COX-2 activity (Doherty and Murray, 2009). This may lead to the development of more refined therapies, with side-effect profiles that allow their generalised use. Previous studies examining gene regulation by COX-2 in CRC cells have focused on long time points and have used relatively high doses of NSAIDs (Zhang and DuBois, 2001). With this in mind, we set out to explore early changes in gene expression in CRC cells resulting from low-dose treatment with a selective COX-2 inhibitor, to improve our understanding of the early signalling events downstream of prostaglandin production. One candidate gene that we have identified, DRAK2 (DAP kinase-related apoptosis-inducing kinase 2 or STK17*β*), is one of a family of serine threonine kinases that share the ability to induce apoptosis (Sanjo *et al*, 1998). The aim of this study was to explore the relationship between COX-2 and DRAK2 as a potential downstream regulator of cell survival in CRC.

## Materials and methods

### Cell culture

All cells were grown in culture at 37°C in a humidified 5% CO_2_ incubator. HCA7 cells were kindly donated by Susan Kirkland (ICRF, London, UK). HCA7 cells were cultured in DMEM with 10% FBS, supplemented with 1 mM sodium pyruvate and 100 *μ*g ml^−1^ kanamycin, to approximately 90% confluence before treatment. HT29 cells were purchased from the ATCC (Rockville, MD, USA) and maintained in McCoy's 5A medium containing 1.5 mM L-glutamine, 10% FBS, penicillin 100 U ml^−1^ and streptomycin 100 *μ*g ml^−1^. SC236, a selective COX-2 inhibitor, was a gift from Dr Peter Isakson (Searle, Skokie, IL, USA). PGE_2_ was purchased from Cayman (St Louis, MO, USA). Staurosporine was purchased from Calbiochem (San Diego, CA, USA). A validated siRNA against a target sequence in exon 3 of the DRAK2 (STK17*β*) gene was purchased from Ambion Inc (Austin, TX, USA). siRNA to scrambled DRAK2 sequence target (scrambled/negative control) was *in vitro* transcribed from oligonucleotide template using Silencer siRNA construction kit (Ambion Inc) . pEGFP-N1 vector was purchased from BD Clontech (San Jose, CA, USA).

### High-density oligonucleotide arrays

Total RNA was isolated from cells and tissue following homogenisation in RNA lysis buffer (Qiagen GmbH, Hilden, Germany) supplemented with 1% *β*-mercaptoethanol. Extraction was performed using RNeasy Midi kits (Qiagen GmbH). RNA quality was determined by agarose gel analysis and RNA concentration was determined by spectrophotometry (GeneQuant pro; Amersham Biotech, Bucks, UK).

Total RNA was isolated as outlined above from HCA7 cells treated for 4, 6 and 8 h with SC236 (5 *μ*M) or vehicle. cDNA was synthesised using the Custom SuperScript ds-cDNA synthesis kit (Invitrogen, Carlsbad, CA, USA). Samples from various time points were pooled and underwent *in vitro* transcription using the ENZO IVT kit (Affymetrix, Santa Clara, CA, USA) to form biotin-labelled cRNA. This was fragmented and three independent biological samples for both control and treated conditions were then hybridised to Affymetrix U95Av2 GeneChips according to Affymetrix protocols. A detailed description of microarray analysis is included as Supplementary methods.

### Quantitative RT-PCR

Total RNA (1 *μ*g) was reverse transcribed using Moloney murine leukaemia virus reverse transcriptase (Promega, Madison, WI, USA) according to the manufacturer's instructions. DRAK2, COX-2 and vascular endothelial growth factor (VEGF) were quantified by RT–PCR using SYBR Green Universal Master Mix (Roche Diagnostics Corp., Indianapolis, IN, USA). Reactions were carried out in a 96-well format in the ABI 7700 Sequence Detector (PerkinElmer/Applied Biosystems, Warrington, Cheshire, UK). Results were then normalised to 18S rRNA amplified from the same cDNA mix and expressed as fold induction compared with the controls. cDNAs were amplified using the following primer pairs: DRAK2, AAAATAGGGCATGCGTGTGAA and TATTATACCAATATTCCACATATCTGTTGCT; COX-2, TTGTACCCGGACAGGATTCTATG and TGTTTGGAGTGGGTTTCAGAAATA; VEGF, CATGCAGATTATGCGGATCAA and TTTGTTGTGCTGTAGGAAGCTCAT. Bax and Bcl-xl quantification was performed using TaqMan pre-developed assay reagents according to the manufacturer's protocol (Applied Biosystems, Foster City, CA, USA).

### Confocal microscopy

HCA7 cells were grown on glass chamber slides and treated with SC236 (5 *μ*M) or vehicle control for 24 h. Slides were air-dried, fixed (methanol) and permeabilised (cold acetone). Slides were then blocked with 1% BSA in PBS (2 h) and incubated with a 1 : 50 dilution of anti-DRAK2 antibody (Santa Cruz Biotech, Santa Cruz, CA, USA) overnight at 4°C. After serial washes, the slides were incubated with a 1 : 250 dilution of fluorescently labelled donkey anti-goat Alexa Fluor 488 (Molecular Probes, Leiden, the Netherlands) and subsequently propidium iodide (Molecular Probes) diluted at 1 *μ*g ml^−1^ in PBS as a nuclear counterstain for 5 min. Slides were mounted with fluorescent mounting media (Dako, Carpinteria, CA, USA) and imaged with an LSM510 Zeiss Axioplan-2 upright confocal microscope (Carl Zeiss, Göttingen, Germany).

### FITC–Annexin V apoptosis assay

HCA7 cells were seeded at a density of 0.5 × 10^6^ cells per well in six-well plates and were treated with SC236 (5 *μ*M) or vehicle control for 24 h. Cells were detached using Versene for 5 min at 37°C and floating and adherent cells were then pelleted by centrifugation and washed in a solution of 2% BSA suspended in PBS. We performed FITC–Annexin V/propidium iodide labelling was performed using a TACS Annexin V–FITC Apoptosis detection kit (R&D Systems, Abingdon, UK). Briefly, cells were re-suspended in a 100 *μ*l working stock of binding buffer, propidium iodide and Annexin V–FITC conjugate and incubated in the dark for 15 min. Cells were re-suspended in binding buffer before analysis in a FACScalibur flow cytometer (Becton Dickinson, Oxford, UK) with measurement of fluorescence emission at 530 nm (FL1 channel) and at >575 nm (FL3 channel). Four quadrant analyses using CellQuest (Becton Dickinson, Oxford, UK) software allowed quantification of cell populations according to labelling characteristics. The results were verified by staining/morphology by confocal microscopy.

### RNA interference and phenotypic characterisation

HT-29 cells were seeded at a density of 0.5 × 10^6^ cells per well in six-well plates and allowed to adhere overnight, then co-transfected with 3 *μ*g of DRAK2 siRNA and 1 *μ*g of pEGFP-N1 (as a marker to select transfected cells) DNA per well using Fugene 6.0 (Roche Diagnostics Corp.) with a 2 *μ*g:1 *μ*l ratio of RNA to transfection reagent. Transfections were performed in OptiMEM, 1 ml per well (final concentration of siRNA, approximately 200 nM). A negative control siRNA to a scrambled target in the *DRAK2* gene (without sequence homology to any other known transcript as verified by BLAST analysis) was used in the mock transfection controls. Populations of both mock- and positively transfected cells were subsequently treated with either SC236 (5 *μ*M), staurosporine (1 *μ*M) or vehicle control for 24 h. Adherent and floating cells were then pelleted, washed and then re-suspended in 250 *μ*l of PBS with 7-aminoactinomycin D (7-ADD; Molecular Probes) at a final concentration of 20 *μ*g ml^−1^ and incubated on ice for 20 min in the dark. Samples were analysed by flow cytometry using an established method for assessment of cell viability in transfected cells selected according to GFP fluorescence (Vezina *et al*, 2001).

### Tumour collection

The protocol was approved by the ethics (medical research) committee of Beaumont Hospital, Dublin and all patients provided written, informed consent. Colon cancer tissue and matched normal colonic mucosa were obtained from patients at the time of surgery and were immediately placed in RNAlater solution (Qiagen GmbH). Total RNA was extracted from the tissue samples as above.

Additional patients with newly diagnosed distal CRCs were recruited under a separate protocol, approved by the Irish Medicines Board and Beaumont Hospital Ethics Committee, for treatment for 5–7 days with the selective COX-2 inhibitor rofecoxib at a dose of 25 mg daily. Tumour was sampled endoscopically at day 0 and again on completion of therapy and was placed in RNAlater. Compliance with drug therapy was monitored by measurement of whole-blood monocyte COX-2 activity as previously described (Panara *et al*, 1995).

### Statistical analysis

Analysis of tumour/normal differences were performed using two-tailed Student's *t*-test. Differences in gene expression across time points were analysed by ANOVA with Bonferroni multiple comparisons test. Linear correlation was assessed using Pearson's test. *t*-Test, ANOVA and correlation statistics were performed using In-Stat, version 3.0 (GraphPad Software, La Jolla, CA, USA).

## Results

### COX-2 modulates susceptibility to apoptosis in HCA7 colon cancer cell line

**Figure 1:**
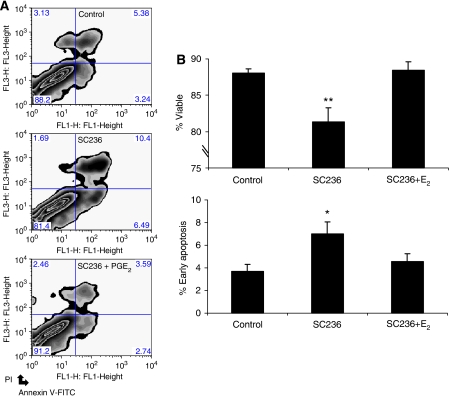
Regulation of HCA7 cell viability and apoptosis by COX-2 inhibition. (**A**) Representative examples of the distribution of staining for FITC–Annexin V (*x* axis, FL1) and propidium iodide (*y* axis, FL3) in HCA7 cells treated for 24 h with vehicle control (top), SC236 (5 *μ*M, middle) and SC236 (5 *μ*M)/PGE_2_ (1 *μ*M, bottom). (**B**) Quantification of cell viability and apoptosis. Viable cells (upper chart) and cells in early stages of apoptosis showing positive Annexin V staining but negative for propidium iodide (lower chart), represented as a percentage of the total cell population. Values shown are mean percentages±s.e.m. of total cell counts across replicate experiments (*n*=4); ^*^P<0.05, ^**^P<0.01; one-way ANOVA. Each replicate involved the counting of a total of a minimum of 10 000 events.

We examined the regulation of apoptosis by COX-2 in a human CRC cell line. HCA7 cells have previously been reported to express high levels of COX-2 with abundant PGE_2_ generation (Sheng *et al*, 1997). Apoptosis was quantified by fluorescence labelling with FITC-conjugated antibody to Annexin V and propidium iodide assessed by flow cytometry (Figure 1). Following a 24 h treatment with the selective COX-2 inhibitor SC236 (5 *μ*M), a significant decrease (*P*=0.01) in HCA7 cells viability was observed (Figure 1B), mirrored by a doubling of the proportion of the cell population gated to the early apoptosis phase (Annexin V positive, propidium iodide negative) (Figure 1B). The observed increase in apoptosis at low micromolar doses of SC236 was prevented by co-administration of exogenous PGE_2_ (1 *μ*M), whereas more marked increases in apoptosis seen with higher doses of SC236 were not rescued. A similar effect was noted with regard to effects on cell proliferation (see Supplementary Figure S1). The ability of PGE_2_ to promote cell survival confirmed that at low micromolar doses the effects of SC236 are largely dependant on its ability to inhibit COX-2.

### High-density oligonucleotide arrays identify SC236-mediated changes in gene expression in HCA7 cells

Having confirmed that COX-2 inhibition causes an apoptotic phenotype in HCA7, we used high-density oligonucleotide microarrays (HDONAs) to examine early global changes in gene expression associated with the abolition of prostaglandin production. Total RNA from cells treated with SC236 (5 *μ*M) or vehicle control for 4, 6 or 8 h was pooled and used to probe Affymetrix HGU95Av2 GeneChips that feature probe sets for over 12 000 different human transcripts. Three independent biological replicates were assayed for each condition (i.e. vehicle control or COX-2 inhibitor).

**Figure 2:**
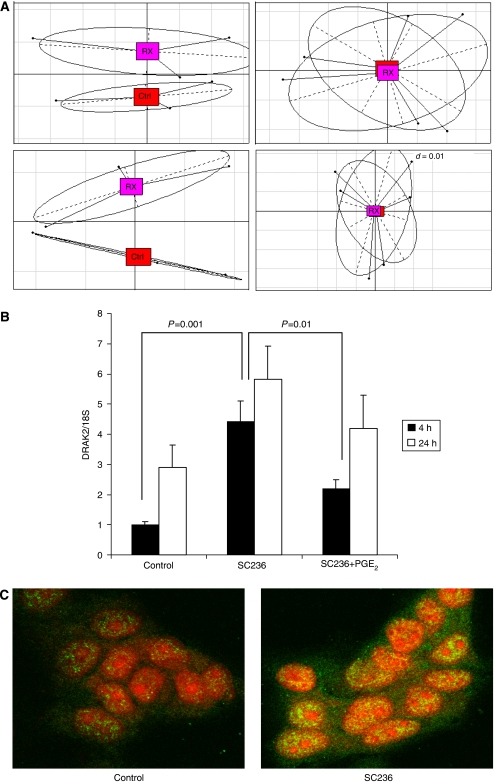
Microarray analysis identifies DRAK2 as one of a number of genes regulated by COX-2 inhibition. (**A**) HCA7 cells display a distinct expression profile following treatment with SC236 (RX, pink) *vs* cells treated with vehicle control (Ctrl, red). The each of four panels illustrates different views of a three-dimensional correspondence analysis (CoA) of RMA-derived expression measures. (**B**) *DRAK2* gene expression in HCA7 cells on treatment with control, SC236 (5 *μ*M) for 4 or 24 h with and without co-treatment with PGE_2_(1 *μ*M). Results of qRT-PCR analysis are expressed as the ratio of *DRAK2* to 18S rRNA. Values shown are the mean±s.e.m. across replicate experiments (*n*=5). (*P*-values are for one-way ANOVA). (**C**) Confocal microscopy of indirect immunofluorescent staining for DRAK2 (green) in HCA7 cells treated with vehicle control or SC236 (5 *μ*M) for 24 h with propidium iodide as a nuclear counterstain (red) (original magnification, × 400).

Robust Multichip Average (RMA)-based analysis was performed to compare expression measures. Magnitude of changes in expression of individual genes was small in most cases, reflecting the early time points used. Correspondence analysis (CoA) of RMA-based measures, however, highlighted significant global differences in gene expression between control and treated samples (Figure 2A). This technique, similar to principal components analysis, represents expression (hybridisation) as points projected in three-dimensional space and demonstrates a consistent alteration in the transcriptome of cells treated with COX-2 inhibitor.

Expression of DRAK2 (STK17*β*) consistently showed strong induction with COX-2 inhibition. This novel serine–threonine kinase is a member of the death-associated protein (DAP) kinase family and like other family members has been implicated in the control of apoptosis (Sanjo *et al*, 1998). However, a potential function in the regulation of cell death and viability in cancer has not been explored to date. A comparison of our expression data set with another recently published HDONA experiment (Levitt *et al*, 2004) (evaluating selective COX-2 inhibition in a breast cell line) revealed a cluster of genes regulated by COX-2 in both systems (see Supplementary data; Table S1) and differential expression of DRAK2 once again featured as a strong signal. It was thus chosen for detailed validation and further analysis.

### Expression of DRAK2 in HCA7 cells is regulated by COX-2 and PGE_2_

Differential DRAK2 expression was confirmed by quantitative RT-PCR (qRT-PCR) on template from HCA7 cells. Induction of DRAK2 expression was observed as early as 4 h after treatment with SC236 and was inhibited by co-administration of PGE_2_ (Figure 2B). A 4.4-fold induction of DRAK2 expression (relative to control) was observed with SC236 (5 *μ*M) after 4 h (*P*=0.001), a response that was attenuated on co-incubation with prostaglandin (SC236 *vs* SC236+PGE_2_, *P*=0.01), suggesting that the changes in DRAK2 expression were dependant on variations in prostaglandin generation. Indirect immunofluorescent staining for DRAK2 in HCA7 cells showed a corresponding increase in levels of DRAK2 protein with COX-2 inhibitor treatment, with development of intense nuclear staining for DRAK2 at 24 h post-treatment with SC236 (Figure 2C).

### Reduced DRAK2 expression in human colorectal tumours

**Figure 3:**
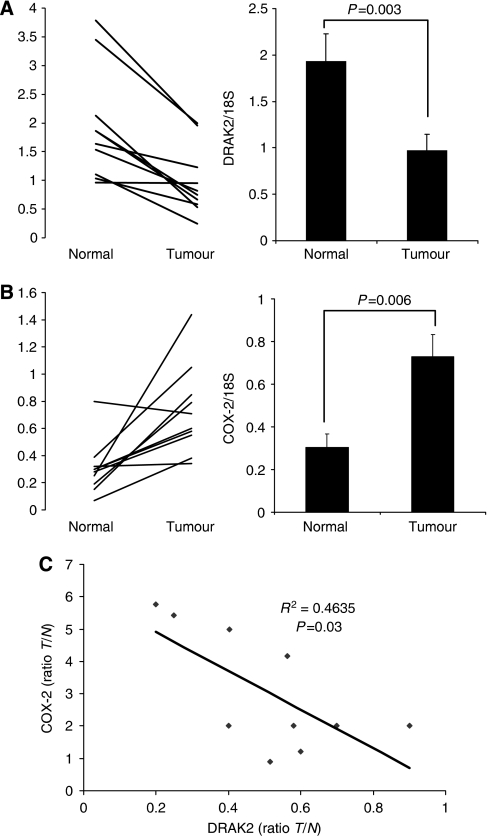
Relationship between expression of DRAK2 and COX-2 in colorectal cancer. (**A**) DRAK2 expression normalised to 18S (as measured by qRT-PCR) in template extracted from normal colorectal mucosa and from colorectal tumour with a line linking the tumour and normal value for each patient (*n*=10). The bar chart shows the mean level of expression in normal and tumour overall (error bars indicate s.e.m., *P*-values for paired Student's *t*-test). (**B**) COX-2 expression normalised to 18S (as measured by qRT-PCR) in the same patient group as above. (**C**) The ratio of COX-2 expression (*y* axis) and DRAK2 expression (*x* axis) in tumour relative to normal for the purposes of linear correlation.

We examined the expression of both DRAK2 and COX-2 in a bank of colorectal tumour samples using total RNA extracted from tumour and normal mucosa sampled from the freshly resected tumours of 10 patients with CRC (not taking aspirin or NSAIDs). The levels of COX-2 transcript (normalised to 18S rRNA) were elevated in the majority of tumour samples relative to normal mucosa (Figure 3B, mean 2.4-fold increase, *P*=0.006). DRAK2 expression showed an opposite pattern, with relative suppression of DRAK2 expression in tumour compared to normal from the same patient (Figure 3A, mean decrease approximately 50%, *P*=0.003). A negative correlation between the ratio of DRAK2 and COX-2 expression (in tumour relative to normal) was noted (Figure 3C, *R*=−0.68, *R*^2^=0.46, *P*=0.03) reflecting an inverse relationship between the tumour/normal differences for the two genes across the patients sampled.

### DRAK2 expression in colorectal tumours is suppressed by COX-2

**Figure 4:**
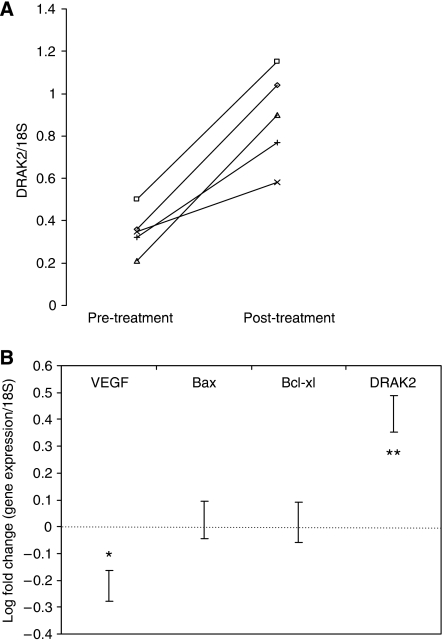
Suppression of DRAK2 expression by COX-2 in colorectal tumours. (**A**) DRAK2 expression normalised to 18S (as measured by qRT-PCR) in template extracted from colorectal tumours sampled before and after treatment with the selective COX-2 inhibitor rofecoxib (with a line joining the pre- and post-treatment values for each patient). (**B**) The log relative fold change in expression (following treatment) of a range of other genes in the same samples for comparison (error bars indicate s.e.m., ^*^P<0.05, ^**^P<0.01; paired Student's *t*-test).

Our observations suggested a suppression of DRAK2 expression, either directly or indirectly, by the actions of COX-2-derived prostaglandins in tumour samples. To test this hypothesis further, five patients with newly diagnosed CRC (not taking aspirin or NSAIDS) were recruited to a pilot study. Endoscopic biopsies of tumour tissue were obtained before and after a short course (5–7 days) of treatment with the COX-2-selective inhibitor rofecoxib at a dose of 25 mg daily, a dose that has been demonstrated to selectively inhibit COX-2 *in vivo* (Ehrich *et al*, 1999). The study was conducted before the withdrawal of rofecoxib by the manufacturer. qRT-PCR demonstrated an induction in DRAK2 expression (Figure 4A; mean 2.5-fold increase, *P*=0.01) in tumour from each of the patients treated with rofecoxib, reflective of the changes seen with COX-2 inhibition *in vitro*. The expression of a panel of additional genes was also evaluated for comparative purposes (Figure 4B). Although no significant change in several genes that modulate apoptosis (Bax, Bcl-xl) was observed, a significant decrease in VEGF expression was noted, with a mean reduction of close to 40% (*P*=0.01). This pro-angiogenic factor has previously been linked to COX-2 activity and reduced VEGF expression has been observed in animal models following treatment with rofecoxib (Yao *et al*, 2003). This finding provided additional validation of the suppression of COX-2 activity in the tumours of these patients.

### DRAK2 silencing by RNAi enhances cell survival with COX-2 inhibition

**Figure 5:**
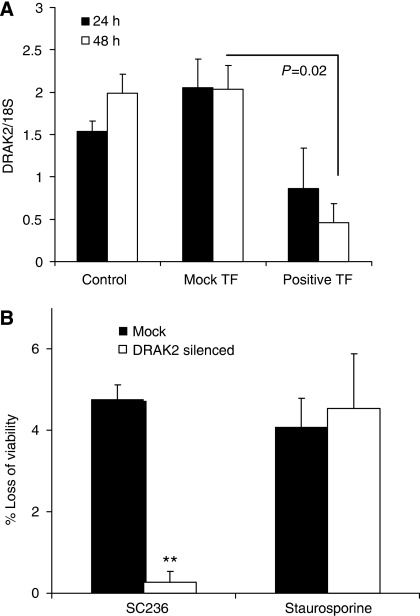
Phenotypic consequences of DRAK2 silencing. (**A**) Effective silencing of DRAK2 expression in HT-29 cells, as measured by qRT-PCR of DRAK2 RNA, following transfection with a DRAK2-specific siRNA for 48 h. Values shown are the mean±s.e.m. (*n*=3); *P*-values, one-way ANOVA. (**B**) Relative reduction in cell viability in mock-transfected (black bars) or DRAK2 siRNA-transfected (white bars) HT29 cells treated with either SC236 (5 *μ*M) or staurosporine (1 *μ*M) for 24 h, compared to untreated control cells. Cells transfected with DRAK2 siRNA were selected by co-transfection of a GFP vector and cell viability was then assessed by uptake of 7-aminoactinomycin D using an established method (Vezina *et al*, 2001). Mean values±s.e.m. (*n*=3); ^*^P<0.05, ^**^P<0.01, one-way ANOVA.

Finally we evaluated the phenotypic activity of DRAK2 in cell systems. We suspected DRAK2 expression was already significantly suppressed in HCA7 cells, given high levels of COX-2 expression whereas HT-29 cells showed lower levels of COX-2 with higher levels of DRAK2 expression. Following transient transfection of HT-29 cells with a commercially available sequence validated siRNA to DRAK2, significant repression of DRAK2 transcription was seen (Figure 5A). To counteract the effects of low transfection efficiency and maximise detection of a phenotype associated with DRAK2 silencing, a GFP expression vector was co-transfected to ‘gate’-transfected or ‘silenced’ cells in subsequent assays to quantify cell survival using an established method (Vezina *et al*, 2001). The percentage of viable cells (without 7-ADD uptake) and non-viable cells (with 7-ADD uptake) was calculated in mock-transfected cells and in DRAK2 siRNA-transfected cells selected (gated) by GFP fluorescence. Loss of cell viability was observed in mock-transfected HT-29 cells following treatment with both SC236 and staurosporine (Figure 5B). In contrast, the siRNA-transfected (GFP-gated) cells showed no loss of cell viability following treatment with the COX-2 inhibitor SC236. They did however still show a significant loss of viability with staurosporine, suggesting a specific effect on the pathway involved in the action of SC236.

## Discussion

Traditional NSAIDs have anti-neoplastic properties, but their prolonged use is limited by their association with gastrointestinal side effects, particularly the risk of gastrointestinal haemorrhage. The new-generation coxibs have also shown promise for chemo-prevention of CRC but are now considered by many to carry an unacceptable risk of thrombotic events. Our aim was to identify novel ‘effector’ pathways operating downstream of COX-2 in tumours, so that this knowledge might allow future selection of more refined therapeutic targets.

There are a number of ways in which COX-2 may promote cancer progression, either through an effect on cancer cells, the associated immune response or angiogenesis in response to the tumour. There is evidence that COX-2 expression protects cells from apoptosis and conversely that treatment of CRC cells with selective COX-2 inhibitors causes cell-cycle arrest, growth inhibition and induction of apoptosis (Richter *et al*, 2001; Sheng *et al*, 1997, 1998). Evidence from both animal models (Hansen-Petrik *et al*, 2002) and human studies (Sinicrope *et al*, 2004) suggests that modulation of cell survival by altered susceptibility to apoptosis is equally important *in vivo*.

However, the precise mechanisms by which COX-2 exerts these effects in tumour cells are unclear. Enhanced COX-2 expression in cells alters susceptibility to tumour necrosis factor-related apoptosis-inducing ligand by reduction in membrane death receptors (Tang *et al*, 2002) and may also alter the threshold for intrinsic pathway activation (Tang *et al*, 2002). Over-expression of COX-2 leads to the generation of prostanoids, particularly PGE_2_, and signalling through the various EP receptors, several of which have been implicated in carcinogenesis (Watanabe *et al*, 1999; Sonoshita *et al*, 2001; Mutoh *et al*, 2002). PGE_2_ stimulates TCF-*β*-catenin-mediated gene transcription (Castellone *et al*, 2005; Shao *et al*, 2005; Eisinger *et al*, 2007), possibly by activation of EP2 and EP4 receptors (Fujino *et al*, 2002). Some of the important effects of PGE_2_ are probably also related to its ability to transactivate the epidermal growth factor receptor through a number of distinct mechanisms (Pai *et al*, 2002; Shao *et al*, 2003; Al-Salihi *et al*, 2007). It seems likely therefore that prostaglandins generated by CRC cells do not exert their activity by a single mechanism but have a range of downstream signalling targets.

We made use of HDONAs to identify ‘early’ target genes downstream of COX-2. Earlier studies in other cell systems have demonstrated the ability of COX-2 to regulate gene expression, such as the expression of MDR1 (Patel *et al*, 2002). Specific studies of differential gene expression in colon cancer cells (using suppressive subtractive hybridisation and differential screening) have previously demonstrated altered expression of genes involved in cell proliferation and viability (Zhang and DuBois, 2001), however high NSAID doses and long time points make a direct comparison with our expression studies difficult. We chose to look at early time points following treatment with the highly selective COX-2 inhibitor SC236 to identify crucial early responses. COX-2 independent effects of SC236 have been described (He *et al*, 2006). However, we chose to use SC236 at a dose which was associated with only modest changes in rates of apoptosis but where we observed rescue by co-incubation with PGE_2_, in the belief that study of a more subtle but specific phenotypic change would yield more significant findings.

We identified DRAK2 as showing consistent changes in expression. This predominantly nuclear serine–threonine kinase has similarity to the DAP kinases and is involved (at least in certain cell types) in initiation of apoptosis (Sanjo *et al*, 1998). Unsupervised hierarchical clustering of our expression data (Supplementary Figure S2) selected a cluster of genes showing a similar pattern of upregulation with COX-2 inhibition. This cluster of genes have either known nuclear localisation within the cell or have a bipartite nuclear localisation signal, which suggests their ability to traffic to the nucleus. Many of these are known to be involved in transcriptional regulation or in the induction or potentiation of apoptosis. Prior expression analyses have previously identified nuclear proteins as significantly over-represented in genes induced by aspirin treatment in colon cancer cells (Hardwick *et al*, 2004) and a mechanism regulating the nuclear trafficking of DRAK2 has recently been suggested (Kuwahara *et al*, 2008).

We have confirmed that DRAK2 expression in colon cancer cells *in vitro* is regulated by COX-2 (Figure 2) and also demonstrated an *in vivo* relationship between COX-2 and DRAK2 expression in human CRC (Figure 3). Both COX-2 and DRAK2 have been implicated in the modulation of T-lymphocyte function, with opposing phenotypic consequences. Activated T cells in patients with SLE markedly upregulate and sustain COX-2 expression and resist inactivation and cell death (Xu *et al*, 2004). Indeed, COX-2 expression has been shown to mediate lethal T-cell activation (Brewer *et al*, 2003). By contrast, over-expression of DRAK2 in activated T cells enhances apoptosis in the presence of IL-2 (Mao *et al*, 2006), and studies in mice with targeted disruption of DRAK2 have demonstrated that this protein is an important negative regulator of T-cell activation, raising the threshold for activation through the T-cell receptor (McGargill *et al*, 2004; Friedrich *et al*, 2005) and acting as an important regulator of T cells response and survival (Schaumburg *et al*, 2007; McGargill *et al*, 2008).

Although it is clear that DRAK2-mediated cell death is cell-type and context dependent, there is a growing body of evidence to indicate that its function is directly related to the regulation of cell survival. In addition to *in vivo* evidence from DRAK2 transgenic mice (Mao *et al*, 2006), induction of DRAK2 *in vitro* in a variety of contexts and cell types augments apoptosis (Sanjo *et al*, 1998; Kuwahara *et al*, 2006; Mao *et al*, 2006). We also show that silencing DRAK2 expression in HT29 cells promotes cell survival and abrogates the effects of a COX-2 inhibitor *in vitro*. These findings confirm published observations on the effect of DRAK2 silencing by RNAi on susceptibility of rat colon cancer cells to UV-induced apoptosis (Kuwahara *et al*, 2006). We also recently observed that DRAK2 may be important in the ability of COX-2 to protect cardiomyocytes from doxorubicin-induced apoptosis (Neilan *et al*, 2006), suggesting that the modulation of DRAK2 expression may not be confined to cancer but may also be important in the positive effects of COX-2 on cell viability and tissue healing in other systems. For the moment, the precise mechanisms by which DRAK2 impacts on cell survival are unclear. Recent observations suggest that the ribosomal kinase p70S6, involved in cell-cycle regulation, is a substrate for DRAK2 (Mao *et al*, 2009), highlighting a possible involvement in regulation of cell-cycle dynamics. In an analysis of publicly available expression data (Whitfield *et al*, 2002), we have noted that DRAK2 forms part of a cluster of genes showing similar cyclical changes in expression with cell-cycle periodicity in HeLa cells (Supplementary Figure S3; Supplementary Table 2). There is an interesting degree of overlap (summarised concisely in Supplementary Figure 4) between this panel and COX-2-regulated genes identified by our gene expression analysis and a number of other gene discovery studies that examined the effects of COX inhibitors (Iizaka *et al*, 2002; Levitt *et al*, 2004).

How does the identification of the regulation of DRAK2 by COX-2 further our understanding of the role of COX-2 in cancer? One of the most consistent observations about COX-2 in cancer is its contribution *in vivo* to the risk of systemic metastases (Tomozawa *et al*, 2000; Yao *et al*, 2004). This fact likely explains the impaired survival observed in individuals whose tumours express COX-2 (Sheehan *et al*, 1999). Previous observations about the effects of DAP kinase (the best-characterised member of the kinase family to which DRAK2 belongs) in cancer cells may provide a unifying explanation, linking the ability of this DAP kinase to regulate apoptosis to a suppression of metastatic potential (Inbal *et al*, 1997). The most plausible explanation is that the enhanced cell survival conferred by DRAK2 silencing (by COX-2) provide a resistance to various forms of cell death and possibly crucially to anoikis (matrix detachment-induced cell death) (Hofmann *et al*, 2007), the form of cell death that may be the most important in deciding the viability of circulating tumour cells and their ability to form distant metastases.
