# Supplementary material for: Regulation of the apoptosis-inducing kinase DRAK2 by cyclooxygenase-2 in colorectal cancer
Source: Br J Cancer. 2009 Jul 28;101(3):483–91. doi: 10.1038/sj.bjc.6605144 (PMC2720240; doi:10.1038/sj.bjc.6605144)
Supplement: Supplementary Figure S1 [file 6605144x1.ppt]

## Slide 1
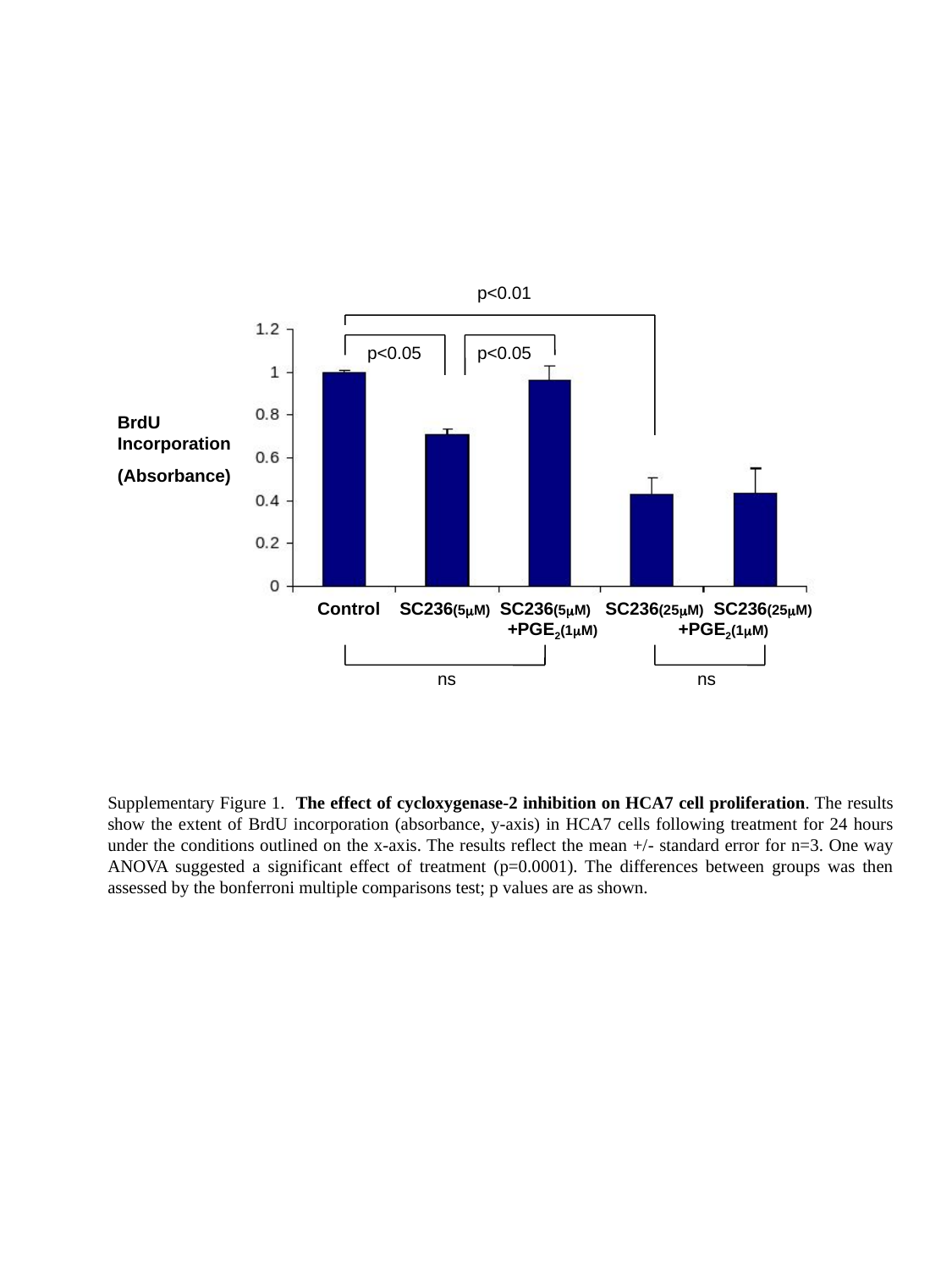

p<0.01
p<0.05
p<0.05
BrdU Incorporation
(Absorbance)
Control SC236(5μM) SC236(5μM) SC236(25μM) SC236(25μM)
+PGE2(1μM)
ns
ns
+PGE2(1μM)
Supplementary Figure 1. The effect of cycloxygenase-2 inhibition on HCA7 cell proliferation. The results show the extent of BrdU incorporation (absorbance, y-axis) in HCA7 cells following treatment for 24 hours under the conditions outlined on the x-axis. The results reflect the mean +/- standard error for n=3. One way ANOVA suggested a significant effect of treatment (p=0.0001). The differences between groups was then assessed by the bonferroni multiple comparisons test; p values are as shown.
