# Supplementary material for: Regulation of the apoptosis-inducing kinase DRAK2 by cyclooxygenase-2 in colorectal cancer
Source: Br J Cancer. 2009 Jul 28;101(3):483–91. doi: 10.1038/sj.bjc.6605144 (PMC2720240; doi:10.1038/sj.bjc.6605144)
Supplement: Supplementary Figure S2 [file 6605144x2.ppt]

## Slide 1
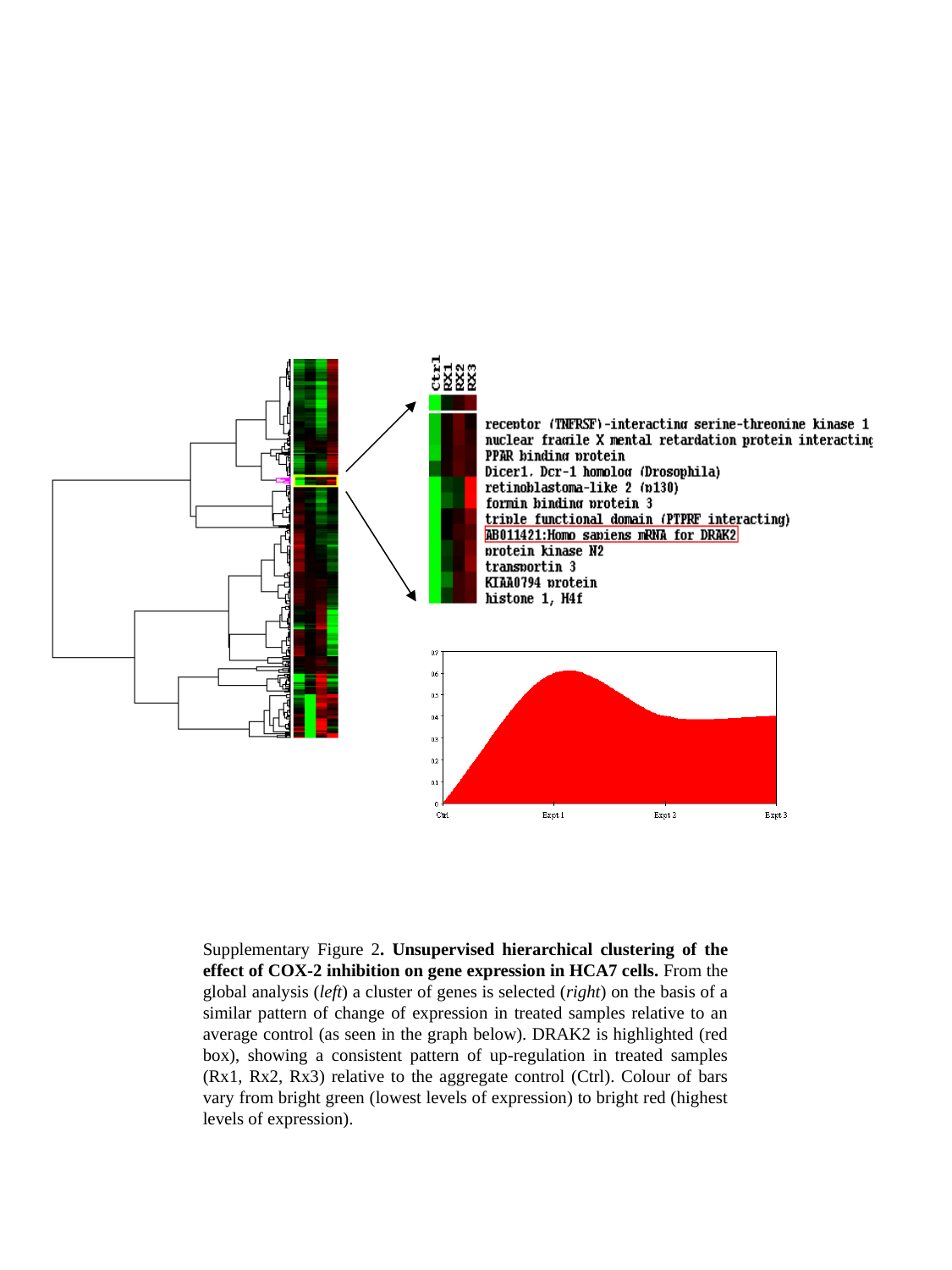

Supplementary Figure 2. Unsupervised hierarchical clustering of the effect of COX-2 inhibition on gene expression in HCA7 cells. From the global analysis (left) a cluster of genes is selected (right) on the basis of a similar pattern of change of expression in treated samples relative to an average control (as seen in the graph below). DRAK2 is highlighted (red box), showing a consistent pattern of up-regulation in treated samples (Rx1, Rx2, Rx3) relative to the aggregate control (Ctrl). Colour of bars vary from bright green (lowest levels of expression) to bright red (highest levels of expression).
