# Supplementary material for: Regulation of the apoptosis-inducing kinase DRAK2 by cyclooxygenase-2 in colorectal cancer
Source: Br J Cancer. 2009 Jul 28;101(3):483–91. doi: 10.1038/sj.bjc.6605144 (PMC2720240; doi:10.1038/sj.bjc.6605144)
Supplement: Supplementary Figure S3 [file 6605144x3.ppt]

## Slide 1
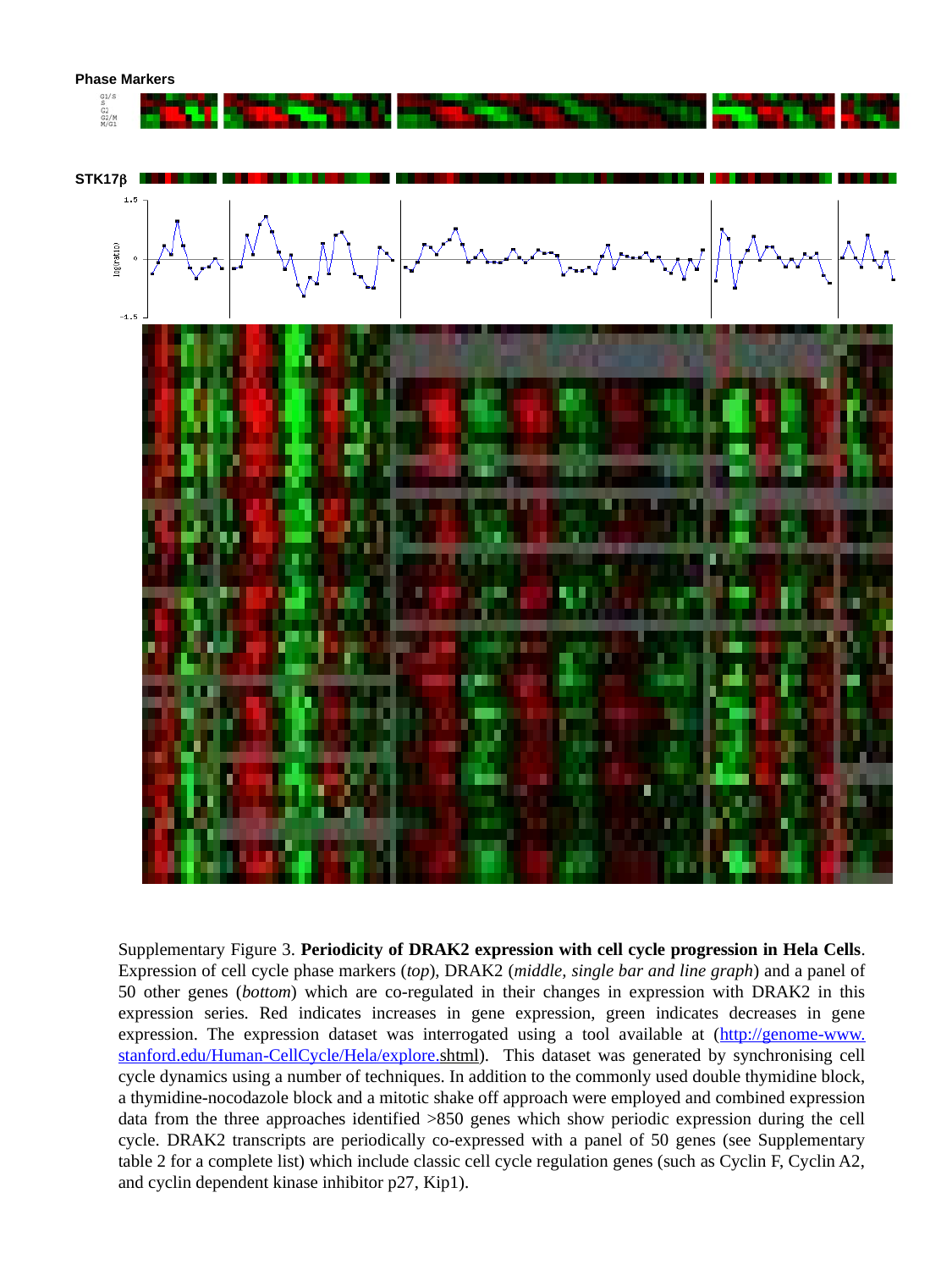

Phase Markers
STK17β
Supplementary Figure 3. Periodicity of DRAK2 expression with cell cycle progression in Hela Cells. Expression of cell cycle phase markers (top), DRAK2 (middle, single bar and line graph) and a panel of 50 other genes (bottom) which are co-regulated in their changes in expression with DRAK2 in this expression series. Red indicates increases in gene expression, green indicates decreases in gene expression. The expression dataset was interrogated using a tool available at (http://genome-www.stanford.edu/Human-CellCycle/Hela/explore.shtml). This dataset was generated by synchronising cell cycle dynamics using a number of techniques. In addition to the commonly used double thymidine block, a thymidine-nocodazole block and a mitotic shake off approach were employed and combined expression data from the three approaches identified >850 genes which show periodic expression during the cell cycle. DRAK2 transcripts are periodically co-expressed with a panel of 50 genes (see Supplementary table 2 for a complete list) which include classic cell cycle regulation genes (such as Cyclin F, Cyclin A2, and cyclin dependent kinase inhibitor p27, Kip1).
