# Supplementary material for: Regulation of the apoptosis-inducing kinase DRAK2 by cyclooxygenase-2 in colorectal cancer
Source: Br J Cancer. 2009 Jul 28;101(3):483–91. doi: 10.1038/sj.bjc.6605144 (PMC2720240; doi:10.1038/sj.bjc.6605144)
Supplement: Supplementary Figure S4 [file 6605144x4.ppt]

## Slide 1
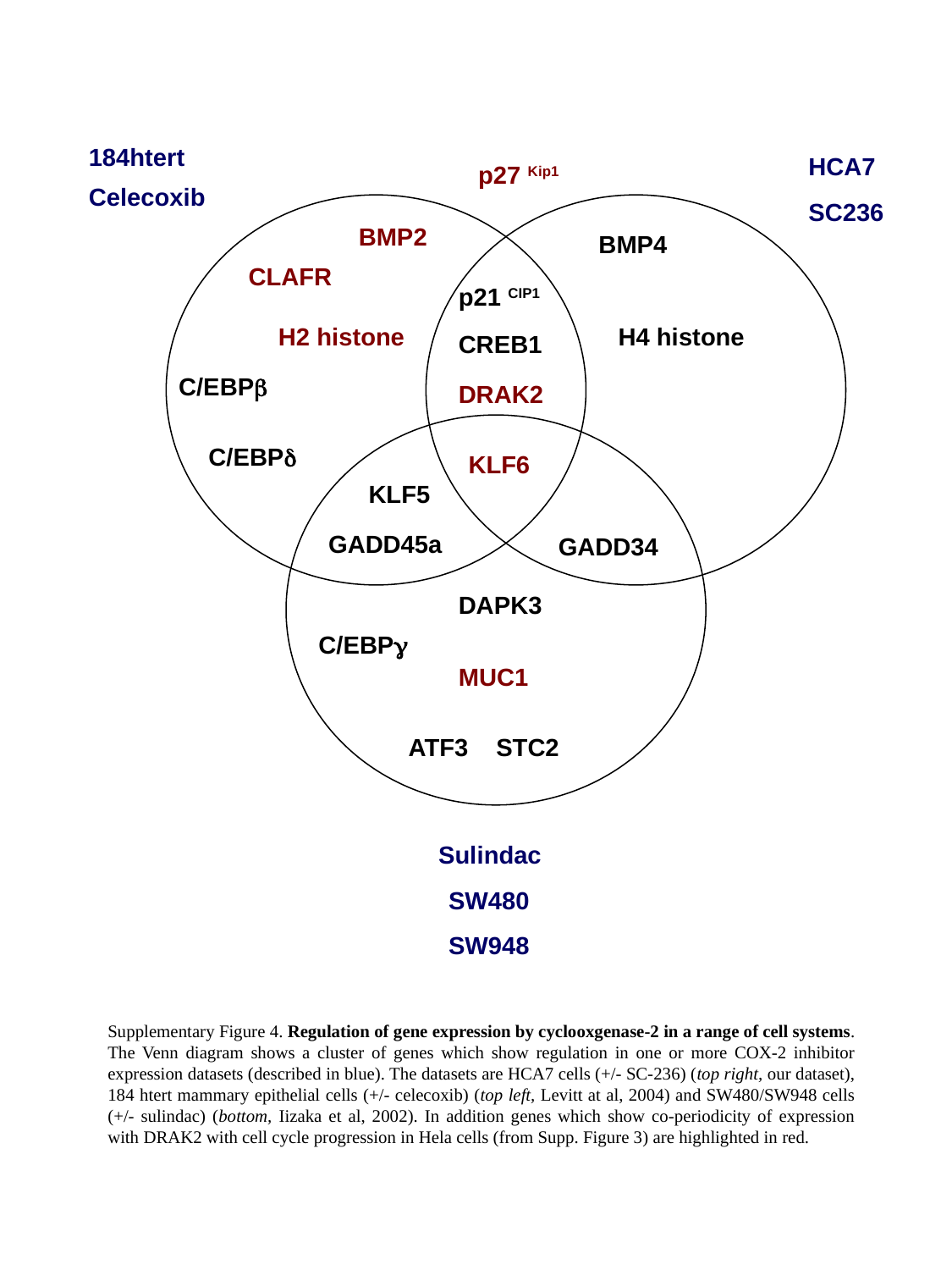

184htert
p27 Kip1
Celecoxib
BMP2
BMP4
CLAFR
p21 CIP1
H2 histone
H4 histone
CREB1
C/EBPβ
DRAK2
C/EBPδ
KLF6
KLF5
GADD45a
GADD34
DAPK3
C/EBPγ
MUC1
ATF3 STC2
Sulindac
SW480
SW948
HCA7
SC236
Supplementary Figure 4. Regulation of gene expression by cyclooxgenase-2 in a range of cell systems. The Venn diagram shows a cluster of genes which show regulation in one or more COX-2 inhibitor expression datasets (described in blue). The datasets are HCA7 cells (+/- SC-236) (top right, our dataset), 184 htert mammary epithelial cells (+/- celecoxib) (top left, Levitt at al, 2004) and SW480/SW948 cells (+/- sulindac) (bottom, Iizaka et al, 2002). In addition genes which show co-periodicity of expression with DRAK2 with cell cycle progression in Hela cells (from Supp. Figure 3) are highlighted in red.
