# Supplementary material for: Regulation of the apoptosis-inducing kinase DRAK2 by cyclooxygenase-2 in colorectal cancer
Source: Br J Cancer. 2009 Jul 28;101(3):483–91. doi: 10.1038/sj.bjc.6605144 (PMC2720240; doi:10.1038/sj.bjc.6605144)
Supplement: Supplementary Table S1 [file 6605144x5.doc]

**Supplementary Table S1**

**Table S1:**

**COX-2 regulated genes in common with Levitt et al dataset**
