# Supplementary material for: Regulation of the apoptosis-inducing kinase DRAK2 by cyclooxygenase-2 in colorectal cancer
Source: Br J Cancer. 2009 Jul 28;101(3):483–91. doi: 10.1038/sj.bjc.6605144 (PMC2720240; doi:10.1038/sj.bjc.6605144)
Supplement: Supplementary Table S2 [file 6605144x6.doc]

Supp Table 2. **Panel of genes showing periodicity of expression with DRAK2 in cell cycle progression in Hela cells**

Gene Symbol Descriptor IMAGE Clone ID

TRIM59  Tripartite motif-containing 59   Clone=IMAGE:303099

Similar to chromosome 15 open reading frame 16; cezanne 2   Clone=IMAGE:278243

KNTC2  Kinetochore associated 2   Clone=IMAGE:345787

HAN11  WD-repeat protein   Clone=IMAGE;1343768

SV2B  Synaptic vesicle glycoprotein 2B   Clone=IMAGE:40893

KIF11  Kinesin family member 11   Clone=IMAGE:825606

DKFZp762E1312  (Hypothetical protein DKFZp762E1312 )  Clone=IMAGE:66406

CCNF  Cyclin F   Clone=IMAGE:455128

CKAP2  Cytoskeleton associated protein 2   Clone=IMAGE:71902

KIAA1712  KIAA1712   Clone=IMAGE:203268

CDCA8  Cell division cycle associated 8   Clone=IMAGE:292936

ESPL1  Extra spindle poles like 1 (S. cerevisiae)   Clone=IMAGE:1416055

KIF23 Kinesin family member 23   Clone=IMAGE:788256

TRIP  TRAF interacting protein   Clone=IMAGE:625584

TUBB  Tubulin, beta polypeptide   Clone=IMAGE:191603

GLI Glioma-associated oncogene homolog (zinc finger protein)   Clone=IMAGE:2030843

FLJ22624  FLJ22624 protein   Clone=IMAGE:824753

C14orf106  Chromosome 14 open reading frame 106   Clone=IMAGE:726588

GAS2L3 Growth arrest-specific 2 like 3   Clone=IMAGE:194656

ARL6IP2  ADP-ribosylation factor-like 6 interacting protein 2   Clone=IMAGE:220395

G2 T56726 (T56726)   Clone=IMAGE:67092

MUC1 Mucin 1, transmembrane   Clone=IMAGE:840687

RNF141  Ring finger protein 141   Clone=IMAGE:299609

MKI67  Antigen identified by monoclonal antibody Ki-67   Clone=IMAGE:510228

KIFC1 Kinesin family member C1   Clone=IMAGE:292933

KLF6 Kruppel-like factor 6   Clone=IMAGE:510381

BMP2 Bone morphogenetic protein 2   Clone=IMAGE:843398

ZMYM1  Zinc finger, MYM domain containing 1   Clone=IMAGE:325150

C15orf20  Chromosome 15 open reading frame 20   Clone=IMAGE:810209

KBTBD2  Kelch repeat and BTB (POZ) domain containing 2   Clone=IMAGE:243727

S phase ESTs, Weakly similar to PC4259 ferritin associated protein Clone=IMAGE:229560

DKFZp762E1312  (Hypothetical protein DKFZp762E1312)  Clone=IMAGE:1540236

CALM2 Calmodulin 2 (phosphorylase kinase, delta)   Clone=IMAGE:347740

C9orf100 Chromosome 9 open reading frame 100   Clone=IMAGE:769944

MGC57827, Similar to RIKEN cDNA 2700049P18 gene  Clone=IMAGE:131316

C9orf100  Chromosome 9 open reading frame 100   Clone=IMAGE:723930

ARHGAP19  Rho GTPase activating protein 19   Clone=IMAGE:773373

IQGAP3  IQ motif containing GTPase activating protein 3   Clone=IMAGE:1862628

AURKB  Aurora kinase B   Clone=IMAGE:245986

H2AFX  H2A histone family, member X   Clone=IMAGE:256664

TOP2A  Topoisomerase (DNA) II alpha 170kDa Clone=IMAGE:301388

CFLAR  CASP8 and FADD-like apoptosis regulator   Clone=IMAGE:813714

HMGB2  High-mobility group box 2   Clone=IMAGE:363103

FZR1  Fizzy/cell division cycle 20 related 1 (Drosophila)   Clone=IMAGE:1056214

MPHOSPH1  M-phase phosphoprotein 1   Clone=IMAGE:292964

CFLAR CASP8 and FADD-like apoptosis regulator   Clone=IMAGE:309776

CDKN1B  Cyclin-dependent kinase inhibitor 1B (p27, Kip1)   Clone=IMAGE:854668

NUSAP1  Nucleolar and spindle associated protein 1   Clone=IMAGE:951241

CCNA2  Cyclin A2   Clone=IMAGE:950690

WSB1  WD repeat and SOCS box-containing 1   Clone=IMAGE:246661
