# Supplementary material for: Regulation of the apoptosis-inducing kinase DRAK2 by cyclooxygenase-2 in colorectal cancer
Source: Br J Cancer. 2009 Jul 28;101(3):483–91. doi: 10.1038/sj.bjc.6605144 (PMC2720240; doi:10.1038/sj.bjc.6605144)
Supplement: Supplementary Methods [file 6605144x7.doc]

**Supplementary Methods - Micro-array Analysis**

Image files were obtained through Affymetrix GeneChip software (MAS5.0). Subsequently robust multichip analysis (RMA) was performed. Image files were obtained through Affymetrix GeneChip software (MAS5.0), and subsequent analysis was performed using Robust Multichip Averages (RMA). RMA is an R-based array analysis technique which does not depend on the expression measures generated by MAS5.0 but analyses directly from the Affymetrix microarray *.cel image file and comprises three steps: background adjustment, quantile normalisation and summarisation ^1^. As each time point was microarrayed in triplicate an average RMA value was computed to ensure that the average was statistically representative and t test and p values were generated. Only those genes with a p value of < 0.01 were included in subsequent bioinformatics analysis. Thereafter, expression data for each time point was compared with control and a signal log ratio of 0.6 or greater (equivalent to a -fold change in expression of 1.5 or greater) was taken to identify significant differential regulation. Using unsupervised hierarchical cluster analysis as described in Eisen and colleagues ^2^ a visual representation of genomic differential expression was attained. Subsequently, correspondence analysis (CoA) was performed according to the technique of Fellenberg ^3^.

**Additional References**

1. Sadlier DM, Connolly SB, Kieran NE, Roxburgh S, Brazil DP, Kairaitis L, Wang Y, Harris DC, Doran P, Brady HR. Sequential extracellular matrix-focused and baited-global cluster analysis of serial transcriptomic profiles identifies candidate modulators of renal tubulointerstitial fibrosis in murine adriamycin-induced nephropathy. J Biol Chem 2004;279:29670-80.

2. Eisen MB, Spellman PT, Brown PO, Botstein D. Cluster analysis and display of genome-wide expression patterns. Proc Natl Acad Sci U S A 1998;95:14863-8.

3. Fellenberg K, Hauser NC, Brors B, Neutzner A, Hoheisel JD, Vingron M. Correspondence analysis applied to microarray data. Proc Natl Acad Sci U S A 2001;98:10781-6.
